# Development-adjusted inequities and temporal trends in rheumatic heart disease burden across ASEAN countries, 2000–2023

**DOI:** 10.3389/fcvm.2026.1860152

**Published:** 2026-08-18

**Authors:** Derren David Rampengan, Muhammad Iqhrammullah, Roy Novri Ramadhan, Michael Hogipranata, Imke Maria Del Rosario Puling, Arga Setyo Setyo Adji, Faqrizal Ria Qhabibi, Radityo Prakoso, Bambang Dwiputra, Ade Meidian Ambari, Muhammad Habiburrahman

**Affiliations:** 1 Faculty of Medicine, Universitas Sam Ratulangi, Manado, Indonesia; 2 Postgraduate School of Public Health, Universitas Muhammadiyah Aceh, Banda Aceh, Indonesia; 3 Faculty of Medicine, Universitas Airlangga, Surabaya, Indonesia; 4 Faculty of Medicine, Universitas Brawijaya, Malang, Indonesia; 5 Faculty of Medicine, Hang Tuah University, Surabaya, Indonesia; 6 National Cardiovascular Centre Harapan Kita, Universitas Indonesia, Jakarta, Indonesia; 7 Division of Pediatric Cardiology and Congenital Heart Disease, Department of Cardiology and Vascular Medicine, National Cardiovascular Centre Harapan Kita, Universitas Indonesia, Jakarta, Indonesia; 8 Division of Interventional Cardiology, Department of Cardiology and Vascular Medicine, National Cardiovascular Centre Harapan Kita, Universitas Indonesia, Jakarta, Indonesia; 9 Division of Cardiovascular Prevention and Rehabilitation, Department of Cardiology and Vascular Medicine, National Cardiovascular Centre Harapan Kita, Universitas Indonesia, Jakarta, Indonesia; 10 Faculty of Medicine, Imperial College London, London, United Kingdom

**Keywords:** ASEAN, burden of disease, global burden of disease, rheumatic heart disease, socio-demographic index, temporal trends

## Abstract

**Background:**

Although health indicators have improved across ASEAN, RHD burden remains uneven and its decline relative to socioeconomic development is unclear. This study aimed to assess temporal trends, burden typologies, and development-adjusted inequities in RHD across ASEAN countries from 2000 to 2023.

**Methods:**

Data from the Global Burden of Disease (GBD) 2023 study including age-standardised rates of RHD-attributable disability-adjusted life years (DALYs), deaths, and prevalence were used for 11 ASEAN countries from 2000 to 2023. Average annual percentage change (AAPC), derived from generalised additive model (GAM) predictions, was used as the primary trend metric to summarise nonlinear trajectories. Estimated annual percentage change (EAPC) was additionally reported as a uniform log-linear measure for cross-country clustering. Socio-demographic Index (SDI) frontier analysis was performed using a GAM with a country-level random-effect term to evaluate development-adjusted performance.

**Results:**

Across ASEAN, the age-standardised RHD DALY rate declined by 41.0%, from 185.20 [95% uncertainty interval (UI): 126.22–260.25] in 2000 to 109.27 (95% UI: 72.17–159.80) per 100,000 in 2023. Age-standardised death rates fell by 46.6%, from 3.42 to 1.83 per 100,000, while prevalence declined only marginally by 2.1%, indicating persistent prevalent burden despite reductions in mortality and DALYs. GAM-derived AAPCs ranged from −1.80% in Timor-Leste to −4.63% in Cambodia, while EAPCs ranged from −0.95% to −4.67%. Joint EAPC–DALY clustering identified Cambodia and Singapore as less concerning, Brunei Darussalam, Indonesia, Malaysia, the Philippines, Thailand, and Viet Nam as moderately concerning, and Lao People's Democratic Republic (PDR), Myanmar, and Timor-Leste as highly concerning. In the SDI-adjusted analysis, the largest higher-than-expected DALY burdens were observed in Myanmar (+80.6 DALYs per 100,000) and Lao PDR (+76.2), whereas Thailand showed the largest lower-than-expected burden (−77.1). In 2023, the highest DALY rates were observed in Lao PDR (298.12 per 100,000), Timor-Leste (281.03), and Myanmar (238.76).

**Conclusion:**

All ASEAN countries made substantial progress in reducing RHD burden, with most showing nonlinear declining trajectories. However, progress remained uneven and major disparities persisted. Countries classified as highly concerning require prioritised investment in primary prevention, early detection, secondary prophylaxis, and long-term RHD care.

## 1 Introduction

Rheumatic heart disease (RHD) represents the most serious sequela of acute rheumatic fever, resulting from an abnormal autoimmune response to group A streptococcal pharyngitis (1). Despite being largely preventable through timely antibiotic treatment of streptococcal throat infection, RHD remains a leading cause of cardiovascular morbidity and mortality in low- and middle-income countries, with an estimated 40.5 million people affected globally in 2015 (2). In 2023, RHD was responsible for over 300,000 deaths and more than 10 million disability-adjusted life years (DALYs) worldwide, with the burden concentrated disproportionately in sub-Saharan Africa, South Asia, and Southeast Asia (3, 4). The epidemiology of RHD is closely intertwined with socioeconomic conditions: poverty, household overcrowding, poor nutrition, and limited access to healthcare collectively amplify the risk of repeated streptococcal infection, impair timely diagnosis, and reduce adherence to secondary prophylaxis, all of which accelerate disease progression to irreversible valvular damage (5, 6). As a result, RHD functions as a sentinel condition for health system inequity, disproportionately affecting children and young adults in settings where primary care access is most limited, and generating a substantial burden of premature disability and death that curtails economic productivity across generations.

The Association of Southeast Asian Nations (ASEAN), comprising eleven member states with a combined population exceeding 680 million, presents a highly heterogeneous health landscape (7). Countries such as Singapore and Malaysia have achieved income levels and health system capacities comparable to high-income nations, whereas Lao People's Democratic Republic (PDR), Myanmar, Cambodia, and Timor-Leste continue to face fundamental challenges in healthcare access and infrastructure (8). This diversity creates a valuable comparative context for examining how socioeconomic development does or does not translate into reductions in preventable cardiovascular disease, though cross-country comparisons of this kind are observational in nature (9). RHD is particularly relevant in this context for two reasons. First, its primary prevention relies on an inexpensive and widely available intervention, specifically benzathine penicillin G for group A streptococcal pharyngitis, making disparities in outcomes a marker of health system access rather than technological limitation (5, 6). Second, the echocardiographic prevalence of RHD substantially exceeds clinically diagnosed cases in endemic settings, suggesting that considerable burden remains undetected and untreated in lower-resource environments (10, 11).

The Socio-demographic Index (SDI), a composite measure of per capita income, mean years of education, and total fertility rate, provides a standardised proxy for national development and health system capacity (12). Prior global burden analyses have shown an inverse relationship between SDI and RHD burden; however, this relationship is nonlinear, and some countries deviate substantially from the expected frontier, either outperforming or underperforming relative to their development level (2, 13). Importantly, socioeconomic development does not automatically translate into equivalent reductions in RHD burden: countries at similar SDI levels may experience vastly different disease burdens depending on the strength of their primary care systems, antibiotic access policies, and rheumatic fever surveillance infrastructure (14). This decoupling of development from disease reduction makes SDI-frontier analysis a particularly powerful tool for identifying countries where health system investments have either successfully exceeded developmental expectations or have failed to keep pace with economic progress.

Although regional burden analyses of RHD exist for sub-Saharan Africa and South Asia, dedicated investigations for ASEAN remain scarce. Existing studies predominantly report pooled regional estimates, obscuring the substantial within-region heterogeneity that is critical for policy planning (15, 16). Furthermore, prior analyses have not systematically applied nonlinear trend modelling or SDI-frontier approaches to characterise typological differences across ASEAN countries. Using the Global Burden of Disease (GBD) 2023 dataset, the present study aimed to (1) characterise temporal trends in RHD burden across 11 ASEAN countries from 2000 to 2023; (2) identify inflection points in national trajectories using generalised additive models; (3) classify countries using joint estimated annual percentage change (EAPC)–DALY k-means clustering based on temporal change and current burden; and (4) quantify each country's development-adjusted performance using an SDI frontier model with a country-level random-effect term. These analyses aim to provide evidence to guide targeted, burden-specific interventions aligned with each country's development trajectory.

## 2 Methods

### 2.1 Study design and data sources

This was a retrospective, analytical study using publicly available data from the GBD Study 2023, provided by the Institute for Health Metrics and Evaluation (IHME) through the Global Health Data Exchange platform (3). The GBD study is a large-scale international collaboration involving over 10,000 researchers from more than 160 countries, providing standardised estimates of disease burden across time, geography, and population (3, 17). Age-standardised rates of RHD-attributable DALYs, deaths, and prevalence per 100,000 population were extracted for 11 ASEAN member states: Brunei Darussalam, Cambodia, Indonesia, Lao PDR, Malaysia, Myanmar, the Philippines, Singapore, Thailand, Timor-Leste, and Viet Nam. Age standardisation was performed by IHME using the GBD World Standard Population. The estimates included all standard GBD age groups, from birth to 95 years and older; therefore, children under five years of age were included within the age-standardised estimates. No minimum age restriction was applied. The present analysis used age-standardised all-age rates only and did not conduct separate age-stratified analyses by paediatric, adult, or elderly age groups.

Data were extracted for both sexes combined and sex-disaggregated estimates for male and female populations from 2000 to 2023. The study period was restricted to 2000–2023 rather than 1990–2023 because GBD estimates for the 1990s carry substantially wider uncertainty intervals in several ASEAN countries, particularly those with limited historical vital registration coverage, sparse cause-of-death data, limited survey evidence, and weaker health information systems during that period. Restricting the analysis to 2000 onwards was intended to improve cross-country comparability and reduce the influence of highly model-dependent early estimates on temporal trend modelling and inflection-point detection. We acknowledge that this approach excludes a historically relevant decade; however, the improved stability and comparability of post-2000 estimates were considered more appropriate for the primary regional analysis. Socio-demographic Index (SDI) values were obtained from the same GBD 2023 dataset for the corresponding years and countries (12). The ASEAN regional aggregate was excluded to avoid duplication and aggregation bias in country-level analyses.

### 2.2 Descriptive burden analysis

For descriptive assessment, burden estimates in 2000 and 2023 were reported for each country and stratified by sex and age standardisation. It is important to note that all burden analyses in this study use age-standardised rates, which are summary measures adjusted to the GBD reference population. The study does not include age-stratified analyses, such as separate estimates for children, working-age adults, or elderly populations, and no age-band-specific or cohort-based analyses were conducted. The percentage change between the two time points was calculated as the relative difference using the following formula:

$$\mathrm{Change}(\text{\%})=[(\mathrm{Valu}e_{2023}-\mathrm{Valu}e_{2000})\;/\;\mathrm{Valu}e_{2000}]\;\times 100$$

This metric was applied to age-standardised prevalence, death rates, and DALY rates, and was reported alongside 95% uncertainty intervals (UIs) derived from the GBD modelling framework.

### 2.3 Temporal trend analysis: EAPC and AAPC

Temporal trends in age-standardised RHD DALY rates were assessed using log-linear regression, in which the natural logarithm of the DALY rate was regressed against calendar year as follows:

$$\ln(\mathrm{DAL}Y_{t})=\alpha +\beta \cdot t+\varepsilon$$

where DALYₜ represents the age-standardised DALY rate in year t, *α* is the intercept, *β* is the slope representing the annual change on the log scale, and *ε* is the error term. The EAPC was derived from the slope coefficient *β* as:

$$\mathrm{EAPC}=100\times (e^{\beta }-1)$$

A negative EAPC indicates a declining trend. The 95% confidence interval (CI) for EAPC was calculated using the corresponding confidence limits of β as:

$$\mathrm{EAPC}(95\text{\%}\mathrm{CI})=100\times (e\hat{\;}\left(\beta \pm 1.96\;\times \;\mathrm{SE}(\beta )\right)-1)$$

where SE(*β*) denotes the standard error of the slope coefficient. Statistical significance was determined at *p* < 0.05. Because preliminary generalised additive model (GAM) analysis demonstrated significant nonlinearity in eight of eleven countries (see Section 2.4), the Average Annual Percentage Change (AAPC) derived from GAM predictions is used as the primary trend metric throughout this study. EAPC was retained for the clustering because it provided a uniform log-linear measure of long-term trend velocity that could be combined consistently with current DALY burden to construct interpretable cross-country policy typologies.

### 2.4 Generalised additive model analysis

To assess whether temporal trends deviated from linearity, GAMs with thin-plate regression splines were fitted to log-transformed DALY rate data for each country using the mgcv package in RStudio version 2026.04.0 (Posit Software, Boston, Massachusetts, United States) (18). The spline basis dimension was set at k = 5 because each country-level time series contained only 24 annual observations from 2000 to 2023. This value was selected as a conservative upper limit to allow limited curvature while reducing the risk of overfitting short and potentially noisy national time series. In mgcv, k defines the maximum basis dimension rather than the final model complexity; the realised smoothness is penalised during model fitting using restricted maximum likelihood (REML). Therefore, the fitted curves should be interpreted as smoothed descriptive trajectories rather than definitive estimates of discrete epidemiological breakpoints.

Goodness-of-fit was evaluated using the coefficient of determination (R^2^) from the corresponding log-linear model. The wide range in country-level R^2^ values, from 0.39 in Timor-Leste to 0.99 in Singapore, likely reflects heterogeneity in population size, data quality, and signal-to-noise ratio across national time series. For Timor-Leste, the low R^2^ and high inter-annual variability are consistent with its small population and greater reliance on modelled GBD estimates with wider uncertainty intervals. Inflection points, defined as years at which the direction or curvature of the trend changed, were identified by detecting sign changes in the second derivative of the GAM-fitted curve. The AAPC was derived from GAM predictions to provide a smoothed summary of the overall trend. Smoothed trajectories and corresponding 95% confidence intervals were visualised based on model predictions.

### 2.5 Country typology classification

Country typology was assessed using joint k-means clustering based on two standardised indicators: the EAPC in age-standardised RHD DALY rates from 2000 to 2023 and the age-standardised DALY rate in 2023. This approach was used to classify countries according to both the pace of temporal change and the remaining current burden. Candidate cluster solutions were evaluated using elbow plots and average silhouette width, with a three-cluster k-means solution selected as the most interpretable and statistically supported classification. K-means clustering was performed after standardisation of both variables using a fixed random seed (set.seed = 123) and 100 random starts. Cluster robustness was assessed using perturbation-based stability analysis with 1,000 iterations and random noise added to the standardised indicators. Given the small number of ASEAN countries, the resulting clusters were interpreted as descriptive policy archetypes rather than definitive latent classes.

### 2.6 SDI frontier analysis

To assess whether each country's RHD burden was higher or lower than expected given its level of socioeconomic development, an SDI frontier analysis was conducted using a generalised additive model with a country-level random-effect term. Age-standardised RHD DALY rates were modelled as a non-linear function of SDI using a penalised regression spline with k = 5, fitted by REML. Because repeated annual observations were available for each country from 2000 to 2023, a country-level random-effect smooth term was included to account for repeated observations and between-country heterogeneity.

The model was specified as:

$$\mathrm{DAL}Y_{i,t}=\beta_{0}+f(\mathrm{SD}I_{i,t})+u_{i}+\varepsilon_{i,t}$$

where uᵢ ∼ N(0, *σ*ᵤ²) and *ε*ᵢ,ₜ ∼ N(0, *σ*²). DALYᵢ,ₜ denotes the age-standardised RHD DALY rate for country i in year t, f(SDIᵢ,ₜ) represents the smooth non-linear function of SDI, uᵢ is the country-level random effect, and *ε*ᵢ,ₜ is the residual error. Model smoothness was estimated using REML. The effective degrees of freedom (EDF) and associated *p*-value were reported for the SDI smooth term after accounting for country-level heterogeneity.

For each country-year observation, the SDI-expected DALY rate was generated from the population-level SDI smooth, excluding the country-specific random-effect term during prediction. The residual performance gap was then computed as:

$$\mathrm{Ga}p_{i,t}=\mathrm{Observed}\;\mathrm{DAL}Y_{i,t}-\mathrm{SDI}\text{-}\mathrm{expected}\;\mathrm{DAL}Y_{i,t}$$

A positive mean gap indicates a higher RHD DALY rate than expected for the country's SDI level, whereas a negative mean gap indicates a lower RHD DALY rate than expected. Country-level performance was summarised by averaging the gap across all years from 2000 to 2023. Development trajectories were visualised by plotting age-standardised RHD DALY rates against SDI over time for each country, with 2000 and 2023 used as the start and end points.

### 2.7 Summary of statistical analysis and data visualization

All statistical analyses were performed using R version 4.4.0 in RStudio version 2026.04.0 (Posit Software, PBC, Boston, Massachusetts, United States). Key packages included dplyr (version 1.2.1), readr (version 2.2.0), and tidyr (version 1.3.2) for data preparation and transformation; readxl (version 1.4.5) for importing spreadsheet data; ggplot2 (version 4.0.3) for visualisation; mgcv (version 1.9-4) for generalised additive modelling; cluster (version 2.1.8.2) for silhouette analysis; ggrepel (version 0.9.8) for plot labelling; broom (version 1.0.12) for extracting regression estimates; scales (version 1.4.0) for axis formatting; sf (version 1.1.1) for spatial data processing; rnaturalearth (version 1.2.0) and rnaturalearthdata (version 1.0.0) for geographic boundary data; and patchwork (version 1.3.2) for combining plots. Statistical significance was defined as a two-sided *p*-value below 0.05. No formal correction for multiple comparisons, such as the Bonferroni correction or false discovery rate, was applied because the analyses were descriptive and exploratory.

### 2.8 Ethical considerations

This study analysed publicly available, anonymised, aggregated country-level estimates derived from the GBD 2023 study. No primary data collection was conducted, and no individual-level, identifiable, or private information was accessed. Formal ethical approval and informed consent were not required in accordance with institutional and national research ethics guidelines and the ethical principles of the Declaration of Helsinki. Regarding the use of artificial intelligence tools in manuscript preparation: generative Artificial intelligence (AI) language tools were used in this study exclusively to assist with grammar editing, language refinement, and sentence clarity in the manuscript text. All statistical analyses, data extraction, analytical decisions, interpretation of results, and intellectual contributions were performed and verified by the human authors. No AI-generated content was introduced into the data, statistical outputs, or reference list without author verification.

## 3 Results

### 3.1 Overall temporal trends in RHD burden, 2000–2023

**Figure 1:**
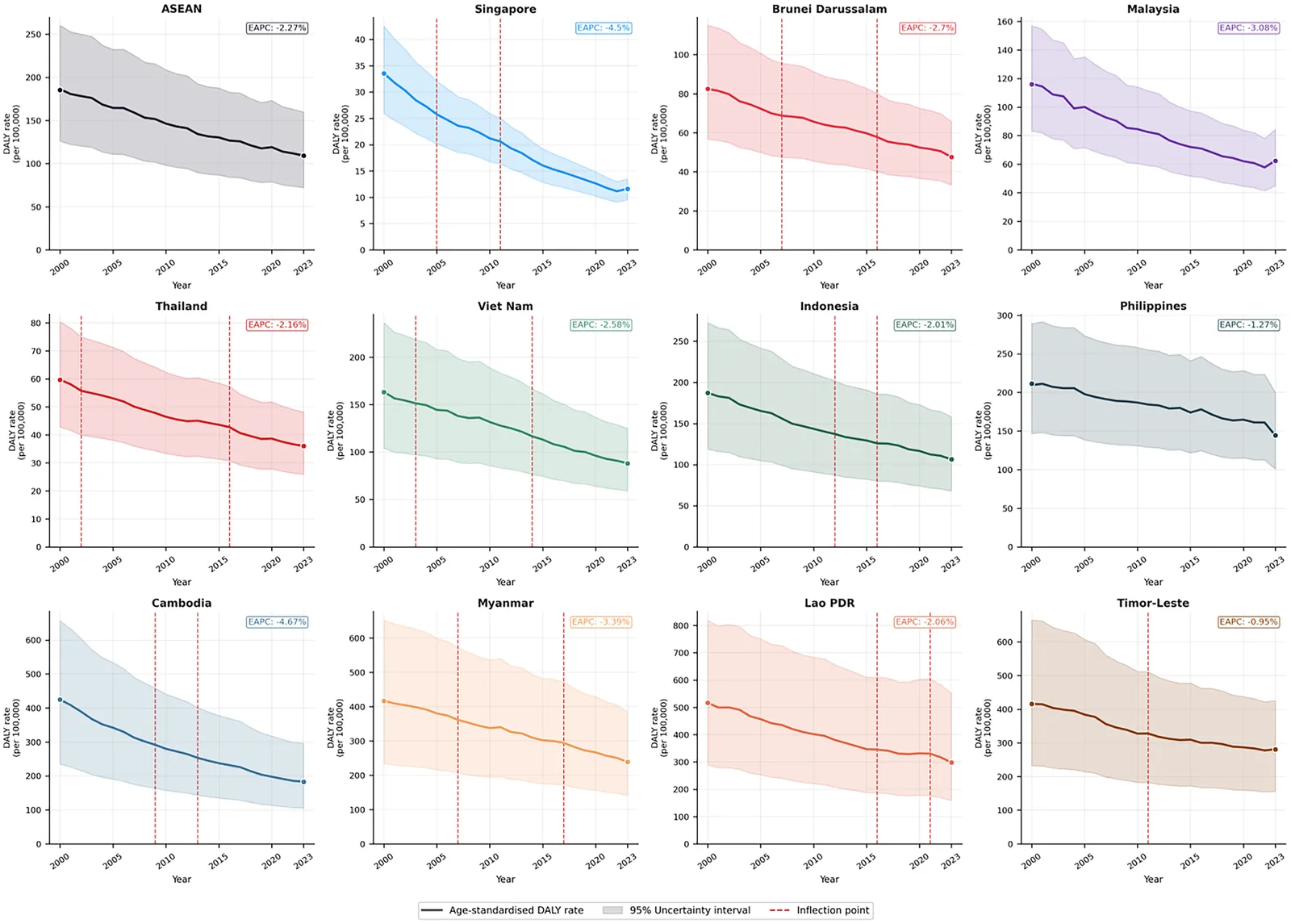
Annual age-standardised rheumatic heart disease DALY rates with 95% uncertainty intervals for ASEAN and member states from 2000 to 2023. Shaded bands represent 95% uncertainty intervals derived from GBD 2023 modelling. DALY, disability-adjusted life year; ASEAN, Association of Southeast Asian Nations; Lao PDR, Lao People's Democratic Republic.

The age-standardised prevalence, death rate, and DALY rates of RHD in ASEAN countries are presented in Table 1, while annual age-standardised RHD DALY trends are shown in Figure 1. From 2000 to 2023, the age-standardised RHD DALY rate across ASEAN declined by 41.0%, from 185.20 (95% UI: 126.22–260.25) to 109.27 (95% UI: 72.17–159.80) per 100,000 population. The age-standardised death rate fell by 46.6%, from 3.42 (95% UI: 2.20–4.86) to 1.83 (95% UI: 1.14–2.87) per 100,000. In contrast, the age-standardised prevalence rate demonstrated only a marginal decline of 2.1%, from 439.41 (95% UI: 349.31–529.14) to 430.14 (95% UI: 342.32–523.39) per 100,000, suggesting that while mortality has improved substantially, the number of people living with RHD has remained relatively stable. The highest DALY burden in 2023 was observed in Lao PDR (298.12 per 100,000; 95% UI: 158.87–551.90), Timor-Leste (281.03; 95% UI: 156.44–425.66), and Myanmar (238.76; 95% UI: 141.13–385.83), more than 25-fold higher than Singapore (11.62; 95% UI: 9.50–13.50). Similarly, death rates in 2023 ranged from 0.35 per 100,000 in Thailand (95% UI: 0.25–0.45) to 5.64 per 100,000 in Lao PDR (95% UI: 2.86–10.80), a 16-fold difference.

**Table 1:** Age-standardised prevalence, death rate, and DALY rate of rheumatic heart disease in ASEAN countries, 2000 and 2023.

Country	Age-standardised prevalence per 100,000 (95% UI)			Age-standardised death rate per 100,000 (95% UI)			Age-standardised DALY rate per 100,000 (95% UI)		
	2000	2023	Change (%)	2000	2023	Change (%)	2000	2023	Change (%)
ASEAN	439.41 (349.31–529.14)	430.14 (342.32–523.39)	−2.11	3.42 (2.20–4.86)	1.83 (1.14–2.87)	−46.58	185.20 (126.22–260.25)	109.27 (72.17–159.80)	−41.00
Brunei Darussalam	41.54 (35.48–48.00)	28.03 (23.39–32.70)	−32.53	2.84 (2.14–3.75)	1.59 (1.04–2.23)	−44.03	62.63 (46.97–84.05)	33.90 (22.71–47.23)	−45.88
Cambodia	414.49 (327.92–509.57)	397.32 (312.87–486.77)	−4.14	9.48 (5.12–14.14)	3.43 (1.87–6.06)	−63.85	512.58 (282.59–756.45)	183.22 (105.23–301.98)	−64.26
Indonesia	449.51 (357.00–542.69)	443.62 (351.29–537.83)	−1.31	2.99 (1.71–4.84)	1.76 (0.91–2.96)	−41.25	166.15 (102.48–257.56)	105.30 (61.16–165.53)	−36.62
Lao PDR	490.31 (391.67–603.67)	483.64 (382.81–598.69)	−1.36	9.40 (5.34–14.25)	5.64 (2.86–10.80)	−40.07	501.94 (286.53–750.81)	298.12 (158.87–551.90)	−40.61
Malaysia	325.75 (264.55–397.29)	302.05 (240.64–370.40)	−7.28	1.55 (1.21–1.99)	0.61 (0.44–0.84)	−60.45	75.44 (59.20–94.14)	38.11 (28.68–48.89)	−49.48
Myanmar	545.86 (433.98–668.08)	529.67 (421.74–656.28)	−2.97	9.57 (4.71–14.08)	4.47 (2.48–7.41)	−53.35	502.17 (254.05–733.27)	238.76 (141.13–385.83)	−52.45
Philippines	410.28 (327.03–494.74)	403.13 (321.35–487.61)	−1.74	2.90 (2.20–3.72)	1.87 (1.33–2.43)	−35.65	153.83 (118.33–195.40)	112.64 (83.89–146.21)	−26.78
Singapore	28.88 (24.70–33.04)	19.56 (16.73–22.97)	−32.25	1.46 (1.25–1.69)	0.59 (0.47–0.71)	−59.50	30.79 (26.59–35.44)	11.62 (9.50–13.50)	−62.26
Thailand	423.33 (338.19–513.06)	419.89 (334.56–514.35)	−0.81	0.86 (0.58–1.24)	0.35 (0.25–0.45)	−59.55	59.71 (42.82–80.34)	36.00 (25.92–48.21)	−39.71
Timor-Leste	538.94 (424.45–662.86)	551.24 (437.43–668.17)	2.28	8.04 (4.42–13.33)	5.14 (2.68–7.91)	−36.12	415.89 (232.06–665.38)	281.03 (156.44–425.66)	−32.43
Viet Nam	451.78 (357.05–552.07)	439.06 (346.79–535.93)	−2.82	3.22 (1.74–4.78)	1.52 (0.86–2.36)	−52.62	163.38 (103.94–236.43)	88.18 (59.07–125.07)	−46.03

Substantial heterogeneity in trend velocity was observed across countries (Tables 2, 3). Based on GAM-derived AAPC, Cambodia recorded the steepest overall decline (AAPC: −4.63%; 95% CI: −5.40 to −3.86), followed by Singapore (AAPC: −4.32%; 95% CI: −5.36 to −3.27) and Myanmar (AAPC: −3.23%; 95% CI: −4.07 to −2.39). Timor-Leste showed the slowest overall decline (AAPC: −1.80%; 95% CI: −2.74 to −0.85), followed by the Philippines (AAPC: −1.23%; 95% CI: −2.31 to −0.14). The ASEAN-level EAPC was −2.27% (95% CI: −2.37 to −2.16; *p* < 0.001); country-level EAPCs are reported in Table 2 for comparability with prior literature.

**Table 2:** Estimated annual percentage change (EAPC) in age-standardised RHD DALY rates in ASEAN countries, 2000–2023.

Country	EAPC (95% CI)	*p*-value
ASEAN	−2.27 (−2.37 to −2.16)	5.85 × 10^−23^
Brunei Darussalam	−2.70 (−2.90 to −2.50)	1.36 × 10^−18^
Cambodia	−4.67 (−5.00 to −4.34)	7.01 × 10^−19^
Indonesia	−2.01 (−2.22 to −1.80)	2.24 × 10^−15^
Lao PDR	−2.06 (−2.46 to −1.65)	5.93 × 10^−10^
Malaysia	−3.08 (−3.34 to −2.81)	3.17 × 10^−17^
Myanmar	−3.39 (−3.91 to −2.87)	5.10 × 10^−12^
Philippines	−1.27 (−1.54 to −1.00)	2.29 × 10^−9^
Singapore	−4.50 (−4.73 to −4.27)	7.40 × 10^−22^
Thailand	−2.16 (−2.63 to −1.69)	3.06 × 10^−9^
Timor-Leste	−0.95 (−1.48 to −0.42)	0.001
Viet Nam	−2.58 (−2.75 to −2.42)	6.62 × 10^−20^

**Table 3:** Generalised additive model analysis of nonlinear temporal trends in age-standardised RHD DALY rates, 2000–2023.

Country	R^2^ (linear)	*p*-value (linear trend)	Inflection points (year)	AAPC (95% CI)	*p*-value (AAPC)
Brunei Darussalam	0.972	<0.001	2007; 2016	−2.60 (−2.93 to −2.28)	<0.001
Cambodia	0.974	<0.001	2009; 2013	−4.63 (−5.40 to −3.86)	<0.001
Indonesia	0.945	<0.001	2012; 2016	−1.85 (−2.59 to −1.10)	<0.001
Lao PDR	0.831	<0.001	2016; 2021	−2.32 (−2.65 to −1.99)	<0.001
Malaysia	0.963	<0.001	None	−3.14 (−4.10 to −2.18)	<0.001
Myanmar	0.890	<0.001	2007; 2017	−3.23 (−4.07 to −2.39)	<0.001
Philippines	0.809	<0.001	None	−1.23 (−2.31 to −0.14)	<0.001
Singapore	0.986	<0.001	2005; 2011	−4.32 (−5.36 to −3.27)	<0.001
Thailand	0.804	<0.001	2002; 2016	−2.35 (−2.87 to −1.83)	<0.001
Timor-Leste	0.386	0.001	2011	−1.80 (−2.74 to −0.85)	<0.001
Viet Nam	0.979	<0.001	2003; 2014	−2.73 (−3.00 to −2.47)	<0.001

### 3.2 Sex-disaggregated trends

Across ASEAN, females consistently bore a higher RHD DALY burden than males throughout the study period. In 2023, the age-standardised DALY rate for females was 134.23 (95% UI: 74.38–217.86) per 100,000, compared to 84.43 (95% UI: 51.90–123.37) for males, representing a cross-sectional female-to-male ratio of approximately 1.59 in that year. This ratio reflects the point-in-time sex differential for 2023 and should not be interpreted as a time-series estimate of sustained excess; temporal sex trends are described separately below. The female excess was most pronounced in countries with high overall burden: in Cambodia, the 2023 age-standardised female DALY rate (217.64 per 100,000) exceeded the male rate (144.77) by 50%. Similar patterns were observed in Indonesia (female: 132.98 vs. male: 78.41 per 100,000) and the Philippines. Changes in age-standardised DALY rates from 2000 to 2023 were comparable between sexes in most countries, with females declining by 40.5% and males by 41.1% across ASEAN. Age-standardised prevalence in 2023 was higher among females (472.65 per 100,000; 95% UI: 376.82–573.54) than males (386.96; 95% UI: 307.71–469.94) across ASEAN. Age-standardised mortality was also higher in females (2.31 per 100,000; 95% UI: 1.13–3.96) compared to males (1.33; 95% UI: 0.74–2.11), consistent with the known differential burden of RHD by sex. Detailed sex-disaggregated estimates for all countries and both time points are summarised in Supplementary Tables S1–S3.

### 3.3 GAM analysis and inflection points

Log-linear models provided a good fit for most countries (R^2^ range: 0.39–0.99), confirming that a substantial proportion of DALY rate variation was explained by temporal trends (Table 3). However, GAM analysis revealed significant nonlinearity in eight of eleven countries, with multiple inflection points identified. Singapore exhibited two inflection points (2005 and 2011), with a steeper rate of decline following 2011. Cambodia showed two inflection points in 2009 and 2013, while Indonesia demonstrated inflections in 2012 and 2016. Further, Viet Nam showed early first inflection point in 2003, and the second one in 2014. Myanmar exhibited inflections in 2007 and 2017, while Thailand showed early acceleration after 2002 followed by renewed improvement after 2016. Malaysia and the Philippines demonstrated highly linear trajectories (R^2^ = 0.96 and 0.81, respectively). Timor-Leste showed a single inflection point in 2011 and the lowest R^2^ value (0.386), reflecting high inter-annual variability likely driven by limited data and small population size.

### 3.4 Country typology

**Figure 2:**
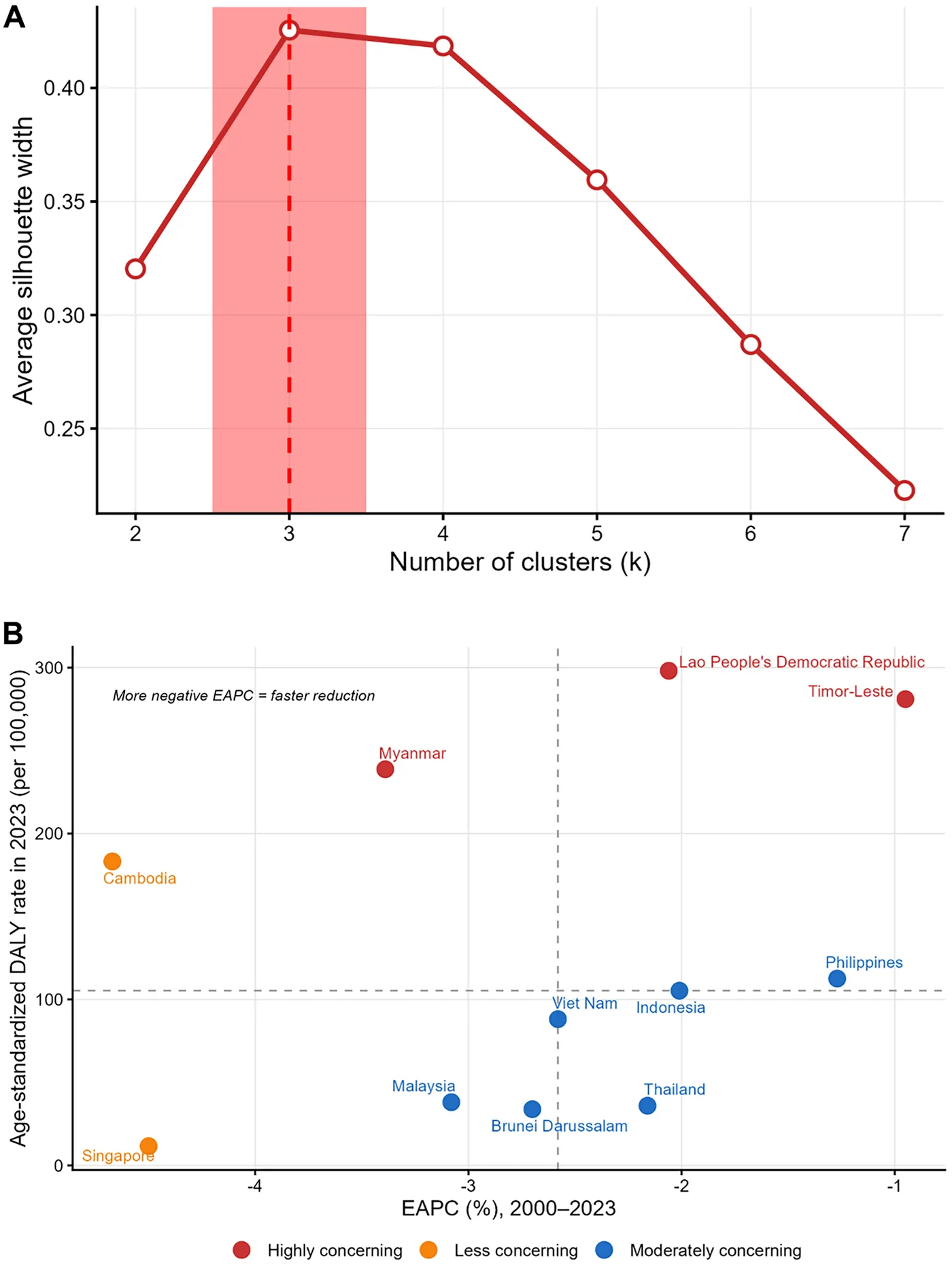
K-means clustering of ASEAN countries based on temporal trends and current rheumatic heart disease burden. Panel A shows the average silhouette width across candidate numbers of clusters, supporting the selection of three clusters (k = 3). Panel B shows the joint clustering of countries according to the estimated annual percentage change (EAPC) in age-standardised RHD DALY rates from 2000 to 2023 and the age-standardised DALY rate in 2023. Colours indicate the resulting concern groups: less concerning, moderately concerning, and highly concerning. EAPC, estimated annual percentage change; DALY, disability-adjusted life year; RHD, rheumatic heart disease; ASEAN, Association of Southeast Asian Nations.

**Figure 3:**
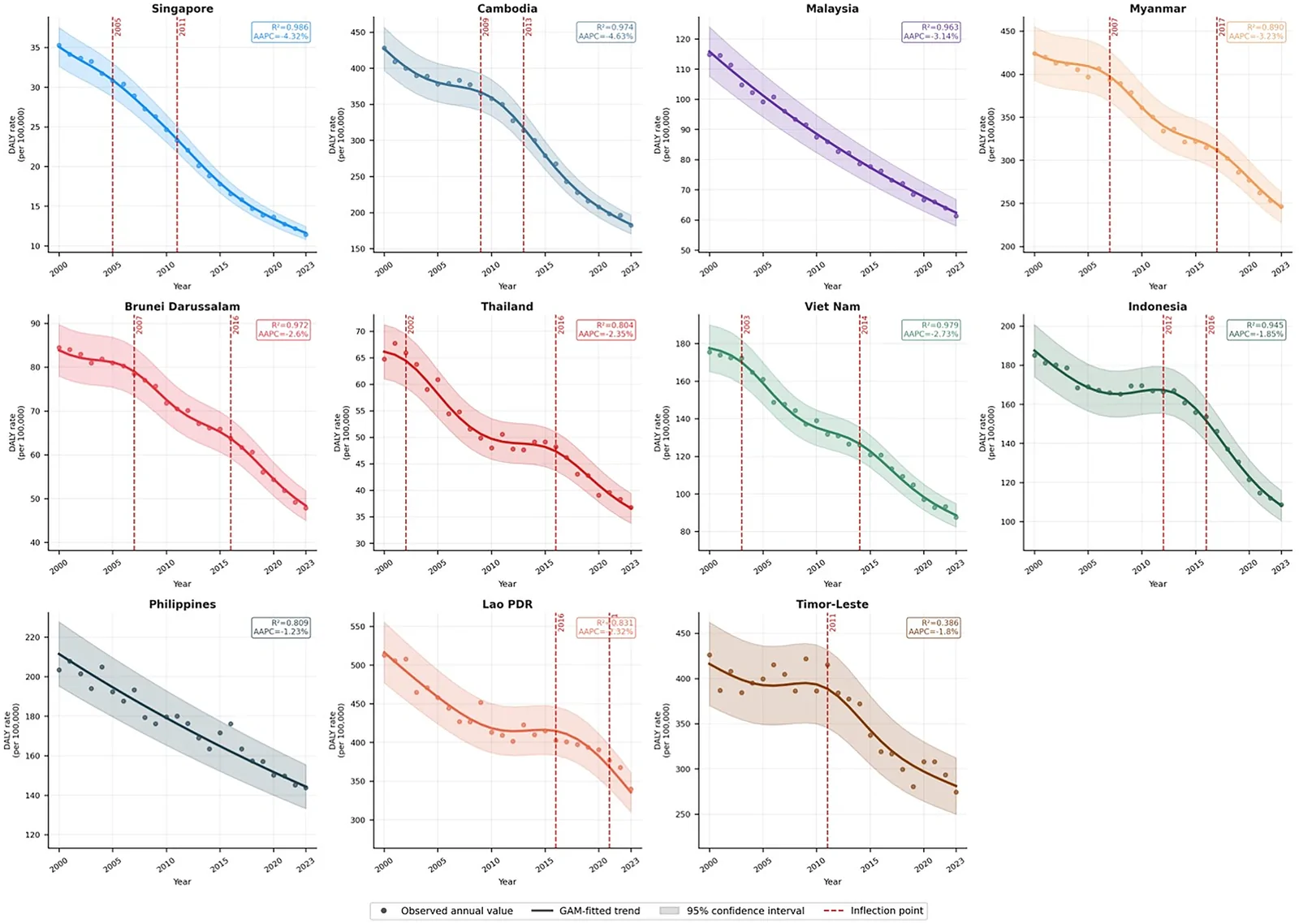
Generalised additive model fitted nonlinear trajectories of age-standardised rheumatic heart disease DALY rates in ASEAN countries, 2000–2023. Points show observed annual values, shaded bands show 95% confidence intervals, and dashed red vertical lines mark identified inflection years. DALY, disability-adjusted life year; Lao PDR, Lao People's Democratic Republic.

Joint k-means clustering based on EAPC and 2023 age-standardised DALY rates identified three country groups (Figure 2). Cambodia and Singapore formed the less concerning cluster. Singapore combined a very low 2023 DALY rate with a strongly negative EAPC, whereas Cambodia showed a comparatively rapid decline despite retaining a moderate residual burden. Brunei Darussalam, Indonesia, Malaysia, the Philippines, Thailand, and Viet Nam formed the moderately concerning cluster, reflecting intermediate combinations of temporal decline and current DALY burden. Lao PDR, Myanmar, and Timor-Leste formed the highly concerning cluster because of persistently high 2023 DALY rates and less favourable joint EAPC–burden profiles. Perturbation-based stability analysis showed generally high assignment stability, although Myanmar showed lower joint-cluster stability, indicating a more borderline classification. Country-specific GAM-fitted trajectories and identified inflection points are shown in Figure 3.

### 3.5 SDI frontier analysis

The SDI frontier model identified a significant nonlinear relationship between SDI and age-standardised RHD DALY rates across ASEAN after accounting for country-level heterogeneity. The SDI smooth term remained statistically significant (EDF = 3.81; *p* < 0.001), while the country-level random-effect term was also significant (EDF = 9.72; *p* < 0.001), indicating substantial between-country heterogeneity beyond the population-level SDI trajectory.

**Figure 4:**
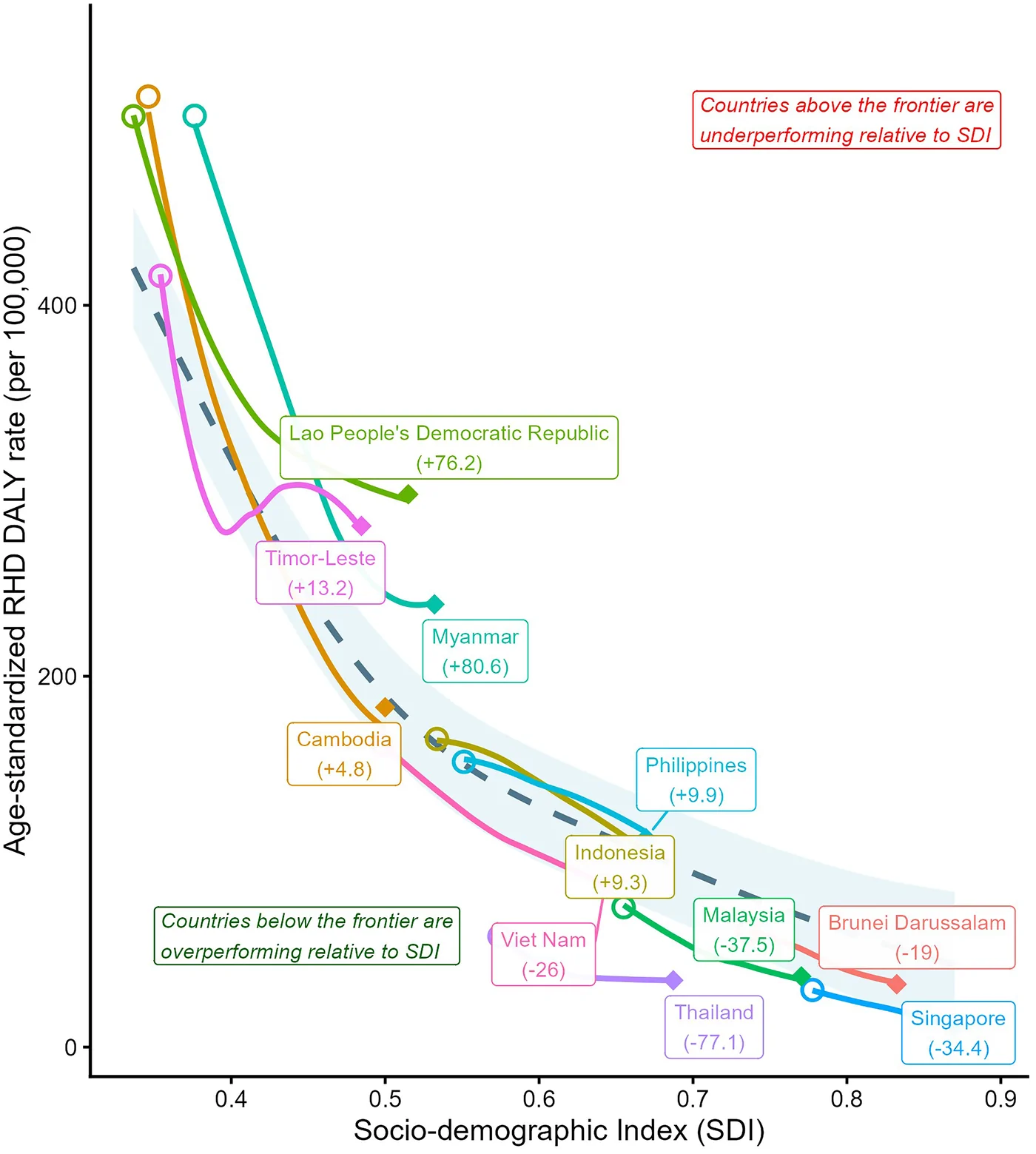
Development trajectories of age-standardised rheumatic heart disease DALY rates relative to the socio-demographic Index across ASEAN countries, 2000–2023. Each coloured curve represents a different ASEAN country, with country identity indicated by the matching colour of its 2023 label. Open circles mark values in 2000, while filled diamonds mark values in 2023. The dashed line represents the population-level DALY rate expected from the generalised additive model after excluding country-specific random effects; the light-blue shaded area represents its 95% confidence interval. Values in parentheses beside country labels are mean observed-minus-expected performance gaps across 2000–2023, expressed as DALYs per 100,000 population. Positive values indicate higher-than-expected burden and relative underperformance, whereas negative values indicate lower-than-expected burden and relative overperformance for the corresponding SDI level. DALY, disability-adjusted life year; RHD, rheumatic heart disease; SDI, socio-demographic index; ASEAN, Association of Southeast Asian Nations.

Performance gaps relative to the SDI-expected trajectory revealed marked development-adjusted disparities. Myanmar had the largest positive mean observed-minus-expected gap (+80.6 DALYs per 100,000), indicating a DALY burden substantially higher than expected for its SDI level, followed by Lao People's Democratic Republic (+76.2). Smaller positive gaps were observed in Timor-Leste (+13.2), the Philippines (+9.9), Indonesia (+9.3), and Cambodia (+4.8). Conversely, Thailand demonstrated the largest negative mean gap (−77.1 DALYs per 100,000), indicating substantially lower burden than expected for its SDI level. Malaysia (−37.5), Singapore (−34.4), Viet Nam (−26.0), and Brunei Darussalam (−19.0) also showed negative gaps, consistent with lower-than-expected RHD DALY rates relative to their development level. Country-specific development trajectories and performance gaps are shown in Figure 4.

## 4 Discussion

This study presents a comprehensive assessment of RHD burden trends, typologies, and SDI-adjusted performance across ASEAN from 2000 to 2023. The findings reveal that while the region has made meaningful progress, with age-standardised DALY rates declining by 41% and death rates by nearly 47%, wide disparities persist, and the trajectory of improvement is neither universal nor automatically guaranteed by economic development alone. The divergence between declining mortality and stable prevalence deserves particular attention. Across ASEAN, the near-static age-standardised prevalence rate, which declined by only 2.1%, contrasted with substantial reductions in DALYs and deaths. This pattern may plausibly reflect improved survival among people living with RHD because of better medical and surgical management, together with increased case detection through expanded use of echocardiographic screening (6, 19). Such a pattern has been described as an epidemiological paradox, whereby successful detection and treatment increase the observable pool of prevalent cases even as mortality declines (20). However, this explanation remains speculative because the present study included neither direct survival data nor information on echocardiographic screening coverage. It should therefore be regarded as a candidate hypothesis requiring confirmation through longitudinal cohort studies or screening-programme registries. These findings nonetheless reinforce the importance of distinguishing prevalence, incidence, mortality, and disability when evaluating the effects of RHD-control programmes.

The country-specific temporal trajectories provide additional insight into how the regional decline evolved. Singapore showed inflection points in 2005 and 2011, which were temporally consistent with the progressive strengthening of rheumatic fever prevention programmes and expanded access to echocardiography, although causal attribution cannot be established from ecological trend data (21). Cambodia showed inflection points in 2009 and 2013, coinciding with the expansion of international health partnerships and primary-care infrastructure, while Indonesia showed inflections in 2012 and 2016 during the phased rollout of the *Jaminan Kesehatan Nasional* (JKN) universal health coverage programme (22, 23). Viet Nam's early inflection in 2003 may plausibly reflect post-renovation gains in health infrastructure and the growing availability of primary care clinics in rural areas, though this association is ecological (24). Thailand's trajectory, with early acceleration after 2002 and renewed improvement after 2016, is temporally consistent with its progressive expansion of the Universal Coverage Scheme and community health worker programme; however, as with all inflection points identified in this study, this temporal co-occurrence cannot establish that programme changes caused the observed trajectory shifts (25). In contrast, Malaysia and the Philippines followed highly linear trajectories, indicating sustained but relatively constant declines without identifiable changes in trend velocity.

The joint clustering analysis identified three country groups with distinct policy implications. Singapore and Cambodia formed the less concerning cluster, although their profiles differed substantially. Singapore represents the lowest-burden benchmark in ASEAN, with an age-standardised DALY rate of 11.62 per 100,000 in 2023 and a strongly negative EAPC, indicating both low current burden and sustained decline. Singapore's long-standing investment in primary care infrastructure and universal health system access is well documented (21), and its echocardiographic screening capacity has been characterised in a prior study (26); however, whether these system-level attributes are the specific drivers of its low RHD burden cannot be established from the present ecological data. Cambodia's inclusion in the same cluster reflects its comparatively rapid decline, despite a remaining DALY rate that is substantially higher than Singapore's. This suggests that Cambodia may represent a country with meaningful recent progress but persistent residual burden, rather than a low-burden benchmark. The pace of Cambodia's decline is consistent with the expansion of non-governmental partnerships, including the World Heart Federation's RHD programme and RHD Action initiative activities, which may have strengthened diagnostic capacity and prophylaxis delivery in resource-constrained settings, although direct programme attribution requires dedicated evaluation data (22, 27).

The highly concerning cluster comprised Lao PDR, Myanmar, and Timor-Leste, all of which retained high age-standardised DALY rates in 2023. This grouping highlights that current residual burden, not only the pace of decline, should guide policy prioritisation. Lao PDR and Myanmar had high residual DALY burdens despite evidence of temporal decline, while Timor-Leste combined high burden with the least favourable EAPC. These profiles suggest persistent gaps in prevention, early detection, secondary prophylaxis, or continuity of RHD care. The moderately concerning cluster, which included Brunei Darussalam, Indonesia, Malaysia, the Philippines, Thailand, and Viet Nam, represents a mixed group in which current burden and decline velocity are intermediate rather than extreme, supporting continued surveillance and targeted strengthening rather than a single uniform intervention strategy.

The SDI frontier analysis provides a development-adjusted benchmark against which country performance may be assessed after accounting for country-level heterogeneity. Myanmar and Lao PDR had the largest positive mean observed-minus-expected gaps (+80.6 and +76.2 DALYs per 100,000, respectively), indicating substantially higher RHD DALY burdens than expected for their SDI levels. In Myanmar, conflict-associated disruption to health service delivery, limited health workforce distribution in rural areas, and historical reliance on out-of-pocket payment may plausibly constrain the conversion of development gains into disease reduction, although these factors have not been formally evaluated in relation to RHD outcomes specifically (28). In Lao PDR, limited published evidence on RHD control capacity, national surveillance programmes, and echocardiography access is consistent with persistent high burden; however, the absence of detailed national health system data on antibiotic prescribing and RHD programme coverage means these potential contributors remain speculative (29).

Thailand showed the largest negative mean performance gap (−77.1 DALYs per 100,000), indicating substantially lower RHD burden than expected for its SDI level. Malaysia (−37.5), Singapore (−34.4), Viet Nam (−26.0), and Brunei Darussalam (−19.0) also showed lower-than-expected DALY rates relative to SDI. Thailand's near-universal health coverage, capitation-funded Universal Coverage Scheme, district-level access to antibiotic treatment, school-based health screening programmes, and community health officer system are contextually consistent with lower-than-expected RHD burden (25). However, formal attribution studies comparing outcomes across health system components would be required to confirm the specific contribution of these policies to Thailand's RHD performance.

Cambodia illustrates why the clustering and SDI-frontier results should be interpreted as complementary rather than interchangeable. Cambodia was classified as less concerning in the joint EAPC–DALY clustering because of its comparatively rapid decline, but its mean SDI-adjusted gap remained slightly positive (+4.8 DALYs per 100,000), indicating that its burden was still marginally higher than expected for its development level. Similarly, Timor-Leste remained above the SDI-expected trajectory (+13.2) and was classified in the highly concerning cluster because of its high residual DALY burden. These findings suggest that rapid progress or programme activity should not be interpreted as full development-adjusted overperformance unless the observed burden is also below the SDI-expected level (27).

**Figure 5:**
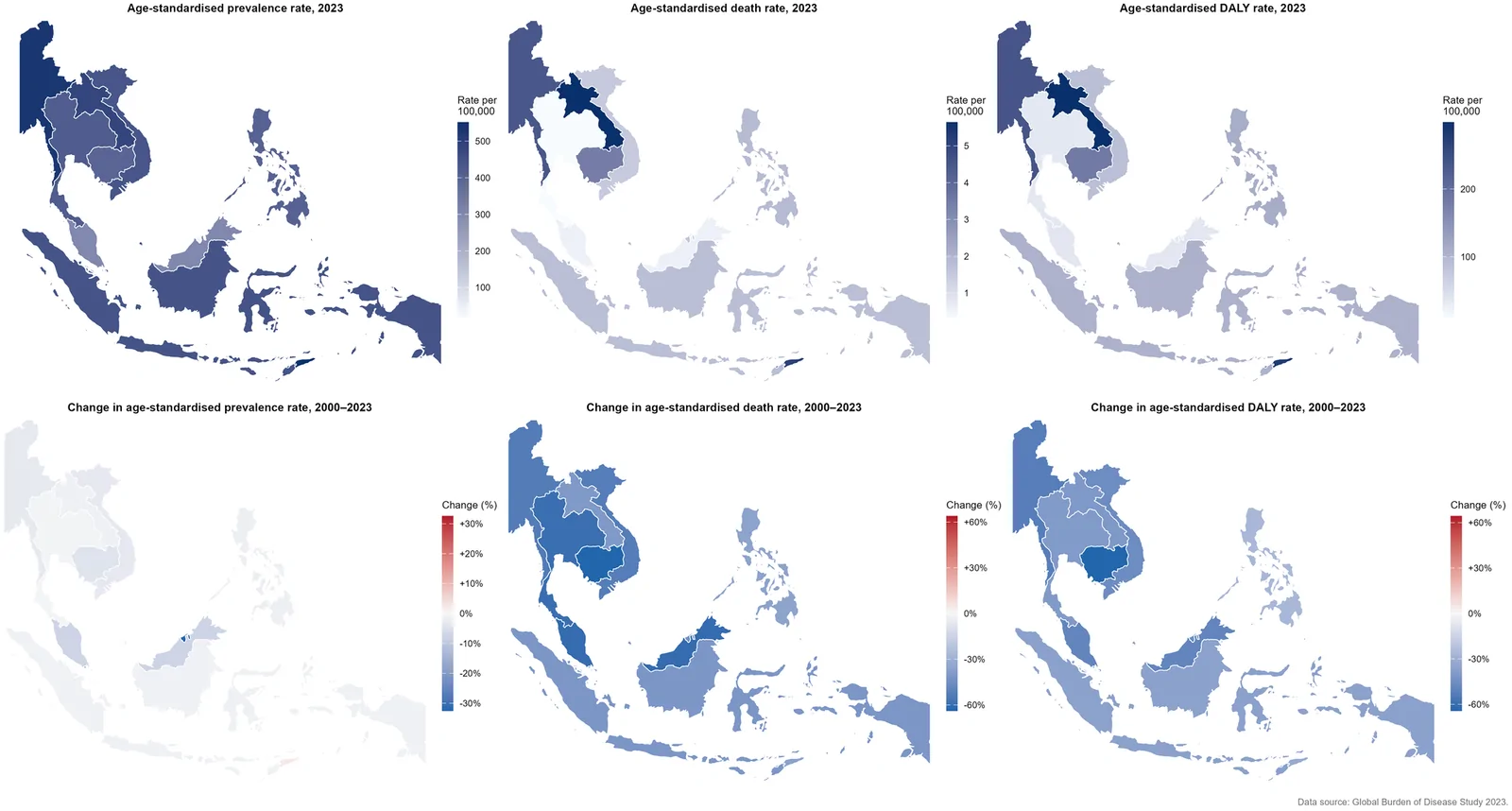
Choropleth maps of age-standardised RHD burden across ASEAN countries, showing 2023 age-standardised prevalence rate, death rate, and DALY rate, together with percentage changes in each metric from 2000 to 2023. In the top row, darker blue indicates higher rates per 100,000 population; in the bottom row, diverging colours indicate percentage change over time.

The female excess in RHD burden observed across ASEAN, with a female-to-male DALY ratio of 1.59, is consistent with global patterns and may reflect differential exposure to streptococcal pharyngitis among girls, the particular susceptibility of the mitral valve to rheumatic damage, and the compounding effects of pregnancy on valvular disease progression (2). These sex-specific risks should be considered in targeting RHD screening and prophylaxis programmes, particularly through antenatal care platforms (30). The geographic distribution of RHD burden across ASEAN showed marked cross-country heterogeneity. The highest age-standardised DALY rates were observed in Lao PDR, Timor-Leste, Myanmar, and Cambodia, whereas substantially lower rates were found in Brunei Darussalam, Malaysia, Singapore, and Thailand. This pattern broadly corresponds to differences in socioeconomic development and health-system capacity. The 2023 age-standardised prevalence, death, and DALY rates, together with percentage changes in each metric from 2000 to 2023 are presented in Figure 5.

### 4.1 Limitations

Several limitations merit acknowledgement. This study relied entirely on the GBD 2023 database as a single secondary data source. The GBD modelling framework incorporates cause-of-death redistribution algorithms, bias adjustments, and disease modelling meta-regression, all of which involve assumptions that may influence point estimates and uncertainty intervals, particularly in countries with sparse or low-quality vital registration data. Although its standardised definitions and modelling procedures improve comparability relative to independently generated national estimates using heterogeneous methods, they cannot completely eliminate systematic differences arising from country-specific input-data availability and quality. The reported uncertainty intervals should therefore not be interpreted as capturing all possible structural or data-source biases. Countries with weaker health information systems, including Timor-Leste, Lao PDR, and Myanmar, are more dependent on modelled rather than direct empirical estimates, potentially reducing the precision and stability of trend estimates and typological classifications in the present study. Differential surveillance coverage, diagnostic ascertainment, and reporting quality may also systematically affect the empirical information available for each country. Migration, forced displacement, and cross-border healthcare utilisation were not explicitly modelled and may influence continuity of prophylaxis, diagnostic ascertainment, and the country in which RHD-related care or deaths are recorded.

The study design is ecological, and temporal correspondence between policy developments and trend inflection points cannot establish causal or programme-specific effects. The policy examples discussed should therefore be interpreted solely as contextual hypotheses requiring evaluation through quasi-experimental studies, programme data, or individual-level longitudinal analyses. Regarding trend metrics, EAPC (derived from log-linear regression) assumes a constant annual rate of change, whereas AAPC (derived from GAM predictions) accommodates nonlinearity. Because GAM analysis demonstrated significant nonlinearity in eight of eleven countries, AAPC should be considered the more appropriate primary trend metric for those countries, and EAPC values for these settings should be interpreted as linear approximations of what are in fact non-constant trajectories. The small numerical discrepancies between EAPC and AAPC values (for example, Lao PDR: −2.06% vs. −2.32%) reflect this methodological distinction rather than analytical inconsistency; both metrics are reported to allow comparability with prior literature that uses EAPC exclusively. Although the SDI frontier model included a country-level random-effect term to account for between-country heterogeneity and repeated observations, it did not explicitly model residual temporal autocorrelation within countries; therefore, confidence intervals and performance gaps should still be interpreted as descriptive comparative indicators rather than causal estimates of health-system performance.

The k-means clustering results depend on the number of clusters specified, which was determined algorithmically but may not uniquely represent true typological boundaries. The multiple statistical tests conducted in this study (twelve EAPCs, eleven GAM nonlinearity tests, eleven SDI gap comparisons) were not subject to formal multiplicity correction; findings should be interpreted as descriptive and exploratory rather than confirmatory. Within-country heterogeneity, including urban-rural and socioeconomic gradients, was not examined in this analysis. Sex-disaggregated country-level estimates are presented in Supplementary Tables S1–S3 and should be interpreted alongside the known reporting differences across GBD 2023 source countries. The primary analyses did not include formal temporal trend modelling of incidence or age-stratified burden estimates. Future analyses incorporating age-stratified data, incidence trend modelling, joinpoint regression, and hierarchical SDI-frontier models would substantially strengthen the epidemiological characterisation of RHD burden across ASEAN.

## 5 Conclusion

RHD burden in ASEAN has declined substantially over two decades, but progress is uneven and disparities persist in the improvement. GAM-derived trend analysis demonstrates that most countries followed nonlinear trajectories, making AAPC a useful summary of smoothed temporal change while EAPC remains important for comparability with prior GBD literature and for the joint EAPC–DALY clustering analysis. Joint clustering identified Lao PDR, Myanmar, and Timor-Leste as the most concerning group because of high residual DALY burden, while the SDI frontier model showed the largest higher-than-expected burdens in Myanmar and Lao PDR. Conversely, Thailand, Malaysia, Singapore, Viet Nam, and Brunei Darussalam had lower-than-expected burdens relative to SDI. These country classifications should be interpreted as descriptive comparative indicators rather than definitive rankings, given differences in underlying data availability and the model-based nature of GBD estimates. Findings from the present study support targeted investments in RHD primary prevention, early detection, echocardiographic access, secondary prophylaxis adherence, and long-term care in countries where burden remains high relative to both temporal progress and level of development. Future analyses incorporating incidence trend modelling, joinpoint regression, temporal autocorrelation structures, health-system covariates, age-stratified burden data, and quasi-experimental designs to evaluate policy effects would further strengthen these findings. Regional cooperation through ASEAN health frameworks, supported by clinical partnerships and shared surveillance infrastructure, offers a strategic mechanism to accelerate convergence and close the RHD equity gap.

## Data Availability

The original contributions presented in the study are included in the article/Supplementary Material, further inquiries can be directed to the corresponding author.
