# Supplementary material for: Development-adjusted inequities and temporal trends in rheumatic heart disease burden across ASEAN countries, 2000–2023
Source: Front Cardiovasc Med. 2026 Aug 18;13:1860152. doi: 10.3389/fcvm.2026.1860152 (PMC13536894; doi:10.3389/fcvm.2026.1860152)
Supplement: Supplementary file 1 [file Datasheet1.docx]

**SUPPLEMENTARY MATERIALS**

**Supplementary Table S1.** Sex-disaggregated prevalence of rheumatic heart disease in ASEAN countries, 2000 and 2023.

| **Country** | **All ages** | | | **Age-standardized** | | |
| --- | --- | --- | --- | --- | --- | --- |
|  | **Value (95% UI)** | | **Change (%)** | **Value (95% UI)** | | **Change (%)** |
|  | **2000** | **2023** |  | **2000** | **2023** |  |
| **ASEAN** |  |  |  |  |  |  |
| Both | 436.38 (345.63 to 532.20) | 463.23 (367.25 to 562.16) | 6.15 | 439.41 (349.31 to 529.14) | 430.14 (342.32 to 523.39) | -2.11 |
| Female | 478.28 (377.41 to 578.75) | 510.75 (406.37 to 616.42) | 6.79 | 480.53 (379.47 to 577.29) | 472.65 (376.82 to 573.54) | -1.64 |
| Male | 394.06 (313.14 to 476.46) | 415.95 (330.02 to 504.02) | 5.56 | 396.11 (314.37 to 477.06) | 386.96 (307.71 to 469.94) | -2.31 |
| **Brunei Darussalam** |  |  |  |  |  |  |
| Both | 21.30 (18.50 to 24.38) | 27.23 (22.71 to 31.71) | 27.83 | 41.54 (35.48 to 48.00) | 28.03 (23.39 to 32.70) | -32.53 |
| Female | 25.92 (22.35 to 29.90) | 34.44 (28.91 to 40.63) | 32.87 | 51.12 (43.10 to 60.55) | 32.99 (27.29 to 38.76) | -35.47 |
| Male | 16.86 (14.41 to 19.57) | 20.79 (17.26 to 24.51) | 23.35 | 32.21 (27.08 to 37.54) | 22.97 (19.34 to 26.74) | -28.69 |
| **Cambodia** |  |  |  |  |  |  |
| Both | 361.55 (287.16 to 443.57) | 408.46 (321.21 to 500.75) | 12.97 | 414.49 (327.92 to 509.57) | 397.32 (312.87 to 486.77) | -4.14 |
| Female | 393.20 (310.52 to 486.42) | 448.44 (349.03 to 557.23) | 14.05 | 441.96 (347.61 to 556.32) | 433.14 (337.36 to 538.25) | -2 |
| Male | 327.85 (251.70 to 402.93) | 366.54 (287.28 to 450.47) | 11.8 | 379.98 (298.30 to 473.91) | 357.96 (282.49 to 440.10) | -5.8 |
| **Indonesia** |  |  |  |  |  |  |
| Both | 451.69 (358.89 to 550.22) | 487.27 (385.16 to 591.61) | 7.88 | 449.51 (357.00 to 542.69) | 443.62 (351.29 to 537.83) | -1.31 |
| Female | 491.93 (387.89 to 600.50) | 530.91 (419.06 to 644.27) | 7.92 | 488.04 (385.80 to 592.84) | 482.14 (381.11 to 583.89) | -1.21 |
| Male | 411.44 (324.52 to 498.51) | 444.73 (351.82 to 537.58) | 8.09 | 409.99 (324.56 to 494.63) | 405.60 (321.81 to 490.19) | -1.07 |
| **Lao PDR** |  |  |  |  |  |  |
| Both | 411.95 (326.41 to 502.04) | 495.62 (390.15 to 616.79) | 20.31 | 490.31 (391.67 to 603.67) | 483.64 (382.81 to 598.69) | -1.36 |
| Female | 435.45 (351.01 to 534.45) | 529.62 (411.34 to 658.52) | 21.62 | 517.93 (415.08 to 639.59) | 517.41 (403.57 to 642.66) | -0.1 |
| Male | 388.22 (304.57 to 472.39) | 462.22 (367.91 to 572.22) | 19.06 | 461.25 (358.33 to 572.29) | 450.07 (356.73 to 558.49) | -2.42 |
| **Malaysia** |  |  |  |  |  |  |
| Both | 316.48 (255.29 to 388.67) | 333.96 (265.41 to 414.07) | 5.52 | 325.75 (264.55 to 397.29) | 302.05 (240.64 to 370.40) | -7.28 |
| Female | 345.38 (278.08 to 424.41) | 369.13 (290.94 to 460.23) | 6.87 | 357.90 (287.80 to 434.52) | 336.58 (266.09 to 416.12) | -5.96 |
| Male | 288.65 (230.01 to 361.58) | 302.16 (238.23 to 377.05) | 4.68 | 294.49 (234.95 to 371.20) | 271.10 (215.12 to 335.44) | -7.94 |
| **Myanmar** |  |  |  |  |  |  |
| Both | 545.79 (431.25 to 660.80) | 542.29 (430.92 to 671.45) | -0.64 | 545.86 (433.98 to 668.08) | 529.67 (421.74 to 656.28) | -2.97 |
| Female | 637.86 (500.50 to 785.56) | 639.13 (503.00 to 796.70) | 0.2 | 633.96 (499.60 to 786.99) | 617.24 (485.88 to 769.86) | -2.64 |
| Male | 448.50 (348.80 to 556.58) | 438.39 (344.15 to 543.66) | -2.25 | 448.85 (347.56 to 555.66) | 431.38 (339.23 to 534.09) | -3.89 |
| **Philippines** |  |  |  |  |  |  |
| Both | 379.01 (301.47 to 458.64) | 412.07 (328.02 to 500.20) | 8.72 | 410.28 (327.03 to 494.74) | 403.13 (321.35 to 487.61) | -1.74 |
| Female | 399.39 (317.48 to 485.24) | 442.14 (350.59 to 536.11) | 10.71 | 436.20 (346.23 to 527.53) | 434.35 (343.95 to 525.82) | -0.42 |
| Male | 359.02 (283.55 to 433.61) | 382.86 (299.67 to 463.79) | 6.64 | 384.05 (303.27 to 461.90) | 371.78 (291.23 to 448.22) | -3.2 |
| **Singapore** |  |  |  |  |  |  |
| Both | 24.87 (21.56 to 28.40) | 29.40 (24.63 to 34.61) | 18.21 | 28.88 (24.70 to 33.04) | 19.56 (16.73 to 22.97) | -32.25 |
| Female | 30.92 (26.31 to 35.66) | 33.19 (27.82 to 39.25) | 7.35 | 33.78 (28.65 to 39.03) | 21.72 (18.41 to 25.41) | -35.71 |
| Male | 19.13 (16.20 to 22.41) | 25.77 (21.32 to 31.40) | 34.71 | 23.69 (20.08 to 27.54) | 17.57 (14.77 to 20.97) | -25.85 |
| **Thailand** |  |  |  |  |  |  |
| Both | 465.61 (370.15 to 569.13) | 475.82 (373.98 to 579.98) | 2.19 | 423.33 (338.19 to 513.06) | 419.89 (334.56 to 514.35) | -0.81 |
| Female | 514.10 (402.49 to 649.66) | 531.90 (410.34 to 655.91) | 3.46 | 462.70 (364.02 to 579.36) | 459.96 (356.47 to 562.26) | -0.59 |
| Male | 415.76 (326.82 to 510.71) | 414.95 (327.88 to 511.54) | -0.2 | 380.64 (300.78 to 465.60) | 375.47 (291.86 to 462.66) | -1.36 |
| **Timor-Leste** |  |  |  |  |  |  |
| Both | 456.75 (356.97 to 561.09) | 506.96 (400.96 to 618.96) | 10.99 | 538.94 (424.45 to 662.86) | 551.24 (437.43 to 668.17) | 2.28 |
| Female | 486.21 (385.12 to 600.33) | 533.99 (415.38 to 656.20) | 9.83 | 574.19 (453.20 to 712.68) | 584.20 (453.53 to 720.71) | 1.74 |
| Male | 428.44 (327.66 to 528.19) | 480.92 (385.27 to 580.79) | 12.25 | 504.27 (388.37 to 628.59) | 519.47 (414.37 to 636.22) | 3.02 |

Supplementary Table S1. Continued.

| **Country** | **All ages** | | | **Age-standardized** | | |
| --- | --- | --- | --- | --- | --- | --- |
|  | **Value (95% UI)** | | **Change (%)** | **Value (95% UI)** | | **Change (%)** |
|  | **2000** | **2023** |  | **2000** | **2023** |  |
| **Viet Nam** |  |  |  |  |  |  |
| Both | 439.12 (346.46 to 537.90) | 475.75 (377.47 to 576.42) | 8.34 | 451.78 (357.05 to 552.07) | 439.06 (346.79 to 535.93) | -2.82 |
| Female | 477.46 (378.04 to 583.83) | 523.47 (416.34 to 647.71) | 9.64 | 490.14 (389.04 to 596.11) | 480.68 (380.91 to 596.84) | -1.93 |
| Male | 399.45 (311.53 to 496.45) | 427.74 (337.92 to 528.45) | 7.08 | 407.88 (318.30 to 506.80) | 395.54 (312.64 to 486.30) | -3.02 |

*Note: Rates are per 100,000 population. Values in parentheses are 95% uncertainty intervals. Change (%) = [(2023 value - 2000 value) / 2000 value] x 100. ASEAN = Association of Southeast Asian Nations; RHD = rheumatic heart disease; UI = uncertainty interval; Lao PDR = Lao People's Democratic Republic.*

**Supplementary Table S2.** Sex-disaggregated mortality of rheumatic heart disease in ASEAN countries, 2000 and 2023.

| **Country** | **All ages** | | | **Age-standardized** | | |
| --- | --- | --- | --- | --- | --- | --- |
|  | **Value (95% UI)** | | **Change (%)** | **Value (95% UI)** | | **Change (%)** |
|  | **2000** | **2023** |  | **2000** | **2023** |  |
| **ASEAN** |  |  |  |  |  |  |
| Both | 3.12 (1.98 to 4.48) | 1.91 (1.19 to 3.01) | -38.65 | 3.42 (2.20 to 4.86) | 1.83 (1.14 to 2.87) | -46.58 |
| Female | 3.94 (2.21 to 6.10) | 2.47 (1.21 to 4.23) | -37.19 | 4.26 (2.45 to 6.51) | 2.31 (1.13 to 3.96) | -45.73 |
| Male | 2.29 (1.25 to 3.73) | 1.35 (0.75 to 2.15) | -40.82 | 2.53 (1.44 to 4.25) | 1.33 (0.74 to 2.11) | -47.33 |
| **Brunei Darussalam** |  |  |  |  |  |  |
| Both | 1.10 (0.82 to 1.49) | 1.17 (0.76 to 1.64) | 5.98 | 2.84 (2.14 to 3.75) | 1.59 (1.04 to 2.23) | -44.03 |
| Female | 1.35 (0.86 to 1.90) | 1.56 (0.83 to 2.50) | 15.6 | 3.44 (2.29 to 4.86) | 1.95 (1.06 to 3.04) | -43.35 |
| Male | 0.86 (0.59 to 1.28) | 0.82 (0.50 to 1.35) | -5.3 | 2.21 (1.52 to 3.28) | 1.16 (0.70 to 1.90) | -47.69 |
| **Cambodia** |  |  |  |  |  |  |
| Both | 8.22 (4.45 to 12.23) | 3.29 (1.80 to 5.78) | -60.02 | 9.48 (5.12 to 14.14) | 3.43 (1.87 to 6.06) | -63.85 |
| Female | 10.49 (5.23 to 16.65) | 4.16 (1.70 to 8.84) | -60.31 | 11.96 (5.90 to 18.81) | 4.29 (1.77 to 9.06) | -64.12 |
| Male | 5.81 (2.43 to 10.80) | 2.37 (1.21 to 4.39) | -59.22 | 6.33 (2.85 to 11.95) | 2.41 (1.22 to 4.42) | -61.96 |
| **Indonesia** |  |  |  |  |  |  |
| Both | 2.73 (1.54 to 4.44) | 1.86 (0.95 to 3.13) | -31.83 | 2.99 (1.71 to 4.84) | 1.76 (0.91 to 2.96) | -41.25 |
| Female | 3.41 (1.44 to 6.75) | 2.46 (0.89 to 4.79) | -27.79 | 3.69 (1.54 to 7.37) | 2.28 (0.83 to 4.43) | -38.35 |
| Male | 2.04 (0.95 to 3.72) | 1.27 (0.53 to 2.16) | -37.85 | 2.29 (1.06 to 4.22) | 1.25 (0.53 to 2.15) | -45.37 |
| **Lao PDR** |  |  |  |  |  |  |
| Both | 7.91 (4.42 to 12.08) | 5.44 (2.74 to 10.45) | -31.28 | 9.40 (5.34 to 14.25) | 5.64 (2.86 to 10.80) | -40.07 |
| Female | 10.13 (4.94 to 17.16) | 7.77 (2.58 to 16.63) | -23.27 | 12.38 (6.08 to 21.07) | 8.00 (2.79 to 17.06) | -35.36 |
| Male | 5.68 (2.57 to 10.37) | 3.15 (1.47 to 5.15) | -44.59 | 6.34 (3.11 to 11.51) | 3.28 (1.50 to 5.38) | -48.28 |
| **Malaysia** |  |  |  |  |  |  |
| Both | 1.16 (0.91 to 1.46) | 0.61 (0.44 to 0.84) | -47.18 | 1.55 (1.21 to 1.99) | 0.61 (0.44 to 0.84) | -60.45 |
| Female | 1.44 (1.00 to 1.99) | 0.80 (0.49 to 1.26) | -44.54 | 1.95 (1.34 to 2.74) | 0.78 (0.47 to 1.24) | -59.96 |
| Male | 0.89 (0.61 to 1.27) | 0.44 (0.29 to 0.65) | -50.04 | 1.14 (0.79 to 1.63) | 0.46 (0.30 to 0.67) | -60.1 |
| **Myanmar** |  |  |  |  |  |  |
| Both | 9.11 (4.41 to 13.43) | 4.41 (2.46 to 7.34) | -51.61 | 9.57 (4.71 to 14.08) | 4.47 (2.48 to 7.41) | -53.35 |
| Female | 10.96 (4.64 to 17.37) | 5.24 (2.28 to 9.60) | -52.24 | 11.19 (4.70 to 18.27) | 5.15 (2.25 to 9.42) | -53.97 |
| Male | 7.14 (2.82 to 12.88) | 3.52 (1.66 to 6.16) | -50.77 | 7.80 (3.22 to 14.19) | 3.72 (1.76 to 6.50) | -52.32 |
| **Philippines** |  |  |  |  |  |  |
| Both | 2.36 (1.77 to 3.03) | 1.80 (1.26 to 2.35) | -24.04 | 2.90 (2.20 to 3.72) | 1.87 (1.33 to 2.43) | -35.65 |
| Female | 2.88 (1.94 to 3.92) | 2.11 (1.28 to 3.03) | -26.74 | 3.54 (2.37 to 4.79) | 2.17 (1.34 to 3.11) | -38.74 |
| Male | 1.86 (1.21 to 3.03) | 1.49 (0.98 to 2.15) | -19.78 | 2.24 (1.49 to 3.72) | 1.56 (1.03 to 2.22) | -30.26 |
| **Singapore** |  |  |  |  |  |  |
| Both | 1.04 (0.89 to 1.20) | 0.95 (0.75 to 1.14) | -8.6 | 1.46 (1.25 to 1.69) | 0.59 (0.47 to 0.71) | -59.5 |
| Female | 1.41 (1.17 to 1.73) | 1.26 (0.93 to 1.61) | -10.55 | 1.71 (1.42 to 2.09) | 0.67 (0.50 to 0.84) | -61.1 |
| Male | 0.68 (0.57 to 0.83) | 0.65 (0.52 to 0.79) | -5.16 | 1.12 (0.94 to 1.36) | 0.49 (0.39 to 0.60) | -56.33 |
| **Thailand** |  |  |  |  |  |  |
| Both | 0.85 (0.57 to 1.22) | 0.45 (0.33 to 0.58) | -46.92 | 0.86 (0.58 to 1.24) | 0.35 (0.25 to 0.45) | -59.55 |
| Female | 1.05 (0.54 to 1.72) | 0.53 (0.33 to 0.74) | -49.76 | 1.05 (0.55 to 1.72) | 0.38 (0.24 to 0.54) | -63.8 |
| Male | 0.64 (0.36 to 1.12) | 0.36 (0.25 to 0.57) | -42.82 | 0.65 (0.37 to 1.11) | 0.31 (0.21 to 0.49) | -52.17 |
| **Timor-Leste** |  |  |  |  |  |  |
| Both | 6.41 (3.35 to 10.51) | 4.60 (2.34 to 7.25) | -28.31 | 8.04 (4.42 to 13.33) | 5.14 (2.68 to 7.91) | -36.12 |
| Female | 9.88 (4.79 to 17.90) | 7.53 (3.15 to 12.84) | -23.76 | 12.50 (6.10 to 23.45) | 8.38 (3.57 to 14.11) | -32.95 |
| Male | 3.08 (1.44 to 5.69) | 1.77 (0.80 to 3.20) | -42.51 | 3.73 (1.79 to 7.44) | 1.98 (0.92 to 3.74) | -46.8 |

Supplementary Table S2. Continued.

| **Country** | **All ages** | | | **Age-standardized** | | |
| --- | --- | --- | --- | --- | --- | --- |
|  | **Value (95% UI)** | | **Change (%)** | **Value (95% UI)** | | **Change (%)** |
|  | **2000** | **2023** |  | **2000** | **2023** |  |
| **Viet Nam** |  |  |  |  |  |  |
| Both | 2.81 (1.55 to 4.24) | 1.69 (0.94 to 2.62) | -39.9 | 3.22 (1.74 to 4.78) | 1.52 (0.86 to 2.36) | -52.62 |
| Female | 3.89 (1.99 to 5.81) | 2.45 (1.20 to 4.05) | -37.15 | 4.31 (2.19 to 6.58) | 2.11 (1.05 to 3.45) | -51.05 |
| Male | 1.68 (0.67 to 3.90) | 0.92 (0.40 to 1.94) | -45.18 | 1.88 (0.78 to 4.35) | 0.89 (0.39 to 1.84) | -52.54 |

*Note: Rates are per 100,000 population. Values in parentheses are 95% uncertainty intervals. Change (%) = [(2023 value - 2000 value) / 2000 value] x 100. ASEAN = Association of Southeast Asian Nations; RHD = rheumatic heart disease; UI = uncertainty interval; Lao PDR = Lao People's Democratic Republic.*

**Supplementary Table S3.** Sex-disaggregated disability-adjusted life years due to rheumatic heart disease in ASEAN countries, 2000 and 2023.

| **Country** | **All ages** | | | **Age-standardized** | | |
| --- | --- | --- | --- | --- | --- | --- |
|  | **Value (95% UI)** | | **Change (%)** | **Value (95% UI)** | | **Change (%)** |
|  | **2000** | **2023** |  | **2000** | **2023** |  |
| **ASEAN** |  |  |  |  |  |  |
| Both | 187.90 (127.17 to 263.93) | 115.15 (75.88 to 168.70) | -38.72 | 185.20 (126.22 to 260.25) | 109.27 (72.17 to 159.80) | -41 |
| Female | 228.49 (137.99 to 346.25) | 142.17 (78.73 to 229.73) | -37.78 | 225.75 (138.49 to 341.10) | 134.23 (74.38 to 217.86) | -40.54 |
| Male | 146.92 (86.01 to 232.84) | 88.28 (54.17 to 129.07) | -39.91 | 143.23 (84.53 to 224.70) | 84.43 (51.90 to 123.37) | -41.05 |
| **Brunei Darussalam** |  |  |  |  |  |  |
| Both | 34.13 (24.62 to 45.56) | 31.62 (20.96 to 44.06) | -7.33 | 62.63 (46.97 to 84.05) | 33.90 (22.71 to 47.23) | -45.88 |
| Female | 39.41 (24.51 to 57.32) | 39.24 (21.29 to 63.28) | -0.42 | 75.79 (48.96 to 106.94) | 40.45 (22.47 to 63.89) | -46.63 |
| Male | 29.04 (19.47 to 42.99) | 24.82 (15.37 to 39.89) | -14.54 | 49.70 (34.25 to 72.75) | 26.63 (16.63 to 43.38) | -46.43 |
| **Cambodia** |  |  |  |  |  |  |
| Both | 500.02 (279.51 to 735.16) | 185.56 (106.76 to 303.59) | -62.89 | 512.58 (282.59 to 756.45) | 183.22 (105.23 to 301.98) | -64.26 |
| Female | 611.78 (321.51 to 970.18) | 218.52 (101.79 to 441.35) | -64.28 | 629.87 (323.52 to 993.83) | 217.64 (101.98 to 438.98) | -65.45 |
| Male | 381.02 (165.55 to 691.34) | 151.01 (82.12 to 259.59) | -60.37 | 369.65 (163.98 to 678.38) | 144.77 (78.91 to 248.85) | -60.84 |
| **Indonesia** |  |  |  |  |  |  |
| Both | 168.48 (102.59 to 259.95) | 113.54 (65.62 to 178.26) | -32.61 | 166.15 (102.48 to 257.56) | 105.30 (61.16 to 165.53) | -36.62 |
| Female | 203.00 (98.20 to 374.93) | 144.49 (65.76 to 259.16) | -28.82 | 201.10 (96.82 to 375.10) | 132.98 (60.18 to 238.16) | -33.87 |
| Male | 133.95 (70.49 to 222.06) | 83.37 (45.11 to 128.78) | -37.76 | 131.09 (70.12 to 220.02) | 78.41 (42.71 to 122.03) | -40.19 |
| **Lao PDR** |  |  |  |  |  |  |
| Both | 482.48 (271.92 to 727.00) | 308.81 (165.10 to 570.33) | -35.99 | 501.94 (286.53 to 750.81) | 298.12 (158.87 to 551.90) | -40.61 |
| Female | 591.99 (297.30 to 983.36) | 427.76 (157.12 to 886.08) | -27.74 | 639.42 (315.30 to 1070.92) | 413.23 (151.02 to 851.83) | -35.37 |
| Male | 371.91 (174.41 to 654.65) | 191.97 (102.39 to 293.85) | -48.38 | 362.10 (173.80 to 647.11) | 184.91 (97.37 to 283.69) | -48.93 |
| **Malaysia** |  |  |  |  |  |  |
| Both | 69.76 (54.23 to 86.36) | 40.91 (30.74 to 52.61) | -41.36 | 75.44 (59.20 to 94.14) | 38.11 (28.68 to 48.89) | -49.48 |
| Female | 80.55 (59.09 to 105.36) | 49.25 (33.11 to 70.73) | -38.86 | 89.24 (65.44 to 117.60) | 45.85 (30.82 to 65.64) | -48.62 |
| Male | 59.37 (42.92 to 82.87) | 33.36 (23.16 to 45.93) | -43.81 | 61.79 (44.19 to 84.24) | 31.09 (21.46 to 42.54) | -49.68 |
| **Myanmar** |  |  |  |  |  |  |
| Both | 524.43 (262.45 to 772.54) | 244.88 (145.09 to 396.61) | -53.3 | 502.17 (254.05 to 733.27) | 238.76 (141.13 to 385.83) | -52.45 |
| Female | 632.94 (298.92 to 990.31) | 290.85 (139.66 to 539.34) | -54.05 | 596.67 (280.56 to 922.47) | 281.38 (135.78 to 521.91) | -52.84 |
| Male | 409.77 (171.34 to 704.20) | 195.56 (101.69 to 323.50) | -52.28 | 400.23 (172.06 to 696.29) | 193.22 (100.92 to 319.69) | -51.72 |
| **Philippines** |  |  |  |  |  |  |
| Both | 147.22 (110.50 to 190.84) | 116.66 (87.16 to 151.58) | -20.76 | 153.83 (118.33 to 195.40) | 112.64 (83.89 to 146.21) | -26.78 |
| Female | 170.45 (119.99 to 230.29) | 129.54 (82.41 to 176.94) | -24 | 181.55 (129.51 to 244.43) | 126.36 (80.38 to 172.49) | -30.4 |
| Male | 124.45 (84.78 to 186.33) | 104.15 (71.10 to 143.33) | -16.31 | 126.07 (87.14 to 192.28) | 99.11 (68.78 to 135.66) | -21.39 |
| **Singapore** |  |  |  |  |  |  |
| Both | 25.75 (22.25 to 29.58) | 17.74 (14.55 to 20.65) | -31.09 | 30.79 (26.59 to 35.44) | 11.62 (9.50 to 13.50) | -62.26 |
| Female | 33.00 (27.30 to 39.48) | 21.89 (16.49 to 27.04) | -33.67 | 36.51 (30.23 to 43.90) | 13.05 (9.66 to 16.06) | -64.26 |
| Male | 18.86 (15.98 to 23.10) | 13.77 (11.18 to 16.38) | -26.99 | 24.06 (20.46 to 29.39) | 10.00 (8.01 to 11.97) | -58.44 |
| **Thailand** |  |  |  |  |  |  |
| Both | 64.13 (45.89 to 86.24) | 39.55 (28.49 to 52.36) | -38.33 | 59.71 (42.82 to 80.34) | 36.00 (25.92 to 48.21) | -39.71 |
| Female | 74.12 (47.70 to 112.88) | 43.58 (30.89 to 60.02) | -41.2 | 68.55 (44.44 to 103.74) | 38.33 (27.32 to 53.39) | -44.08 |
| Male | 53.86 (34.98 to 83.89) | 35.18 (24.73 to 47.89) | -34.69 | 50.08 (32.71 to 77.65) | 33.30 (23.73 to 45.61) | -33.51 |
| **Timor-Leste** |  |  |  |  |  |  |
| Both | 393.36 (212.98 to 615.48) | 277.28 (153.18 to 420.55) | -29.51 | 415.89 (232.06 to 665.38) | 281.03 (156.44 to 425.66) | -32.43 |
| Female | 584.05 (293.65 to 999.39) | 436.40 (198.27 to 716.53) | -25.28 | 628.23 (322.55 to 1135.24) | 443.18 (204.05 to 736.78) | -29.46 |
| Male | 210.07 (111.91 to 356.64) | 124.04 (64.07 to 197.56) | -40.95 | 209.48 (110.18 to 369.13) | 123.56 (65.82 to 197.54) | -41.01 |

Supplementary Table S3. Continued.

| **Country** | **All ages** | | | **Age-standardized** | | |
| --- | --- | --- | --- | --- | --- | --- |
|  | **Value (95% UI)** | | **Change (%)** | **Value (95% UI)** | | **Change (%)** |
|  | **2000** | **2023** |  | **2000** | **2023** |  |
| **Viet Nam** |  |  |  |  |  |  |
| Both | 159.05 (102.88 to 230.25) | 96.05 (63.97 to 136.01) | -39.61 | 163.38 (103.94 to 236.43) | 88.18 (59.07 to 125.07) | -46.03 |
| Female | 203.82 (121.34 to 293.81) | 127.69 (75.54 to 191.01) | -37.35 | 209.89 (122.42 to 301.27) | 115.64 (68.99 to 171.29) | -44.91 |
| Male | 112.71 (56.13 to 229.11) | 64.20 (37.23 to 112.43) | -43.04 | 110.57 (55.06 to 228.32) | 60.22 (34.79 to 104.18) | -45.53 |

*Note: Rates are per 100,000 population. Values in parentheses are 95% uncertainty intervals. Change (%) = [(2023 value - 2000 value) / 2000 value] x 100. ASEAN = Association of Southeast Asian Nations; RHD = rheumatic heart disease; UI = uncertainty interval; Lao PDR = Lao People's Democratic Republic.*

**Supplementary Table S4.** Joint k-means cluster assignments and perturbation stability based on EAPC and age-standardised RHD DALY rates in 2023.

| **Country** | **EAPC (%)** | **DALY rate 2023** | **Concern group** | **Assignment stability** |
| --- | --- | --- | --- | --- |
| Brunei Darussalam | -2.70 | 33.90 | Moderately concerning | 1.000 |
| Cambodia | -4.67 | 183.22 | Less concerning | 1.000 |
| Indonesia | -2.01 | 105.30 | Moderately concerning | 0.990 |
| Lao PDR | -2.06 | 298.12 | Highly concerning | 1.000 |
| Malaysia | -3.08 | 38.11 | Moderately concerning | 0.984 |
| Myanmar | -3.39 | 238.76 | Highly concerning | 0.516 |
| Philippines | -1.27 | 112.64 | Moderately concerning | 0.954 |
| Singapore | -4.50 | 11.62 | Less concerning | 0.948 |
| Thailand | -2.16 | 36.00 | Moderately concerning | 1.000 |
| Timor-Leste | -0.95 | 281.03 | Highly concerning | 1.000 |
| Viet Nam | -2.58 | 88.18 | Moderately concerning | 1.000 |

*Note: EAPC = estimated annual percentage change; DALY = disability-adjusted life year; RHD = rheumatic heart disease. Assignment stability represents the proportion of 1,000 perturbation iterations in which each country retained its original joint k-means cluster assignment. Countries were clustered using standardised EAPC and 2023 age-standardised RHD DALY rates.*
